# Effects of *Arctium lappa L.* Root Powder on Some Markers of Oxidative Stress and Inflammation in Women with Polycystic Ovary Syndrome: A Randomized, Double-Blind Controlled Clinical Trial Study

**DOI:** 10.5935/1518-0557.20250189

**Published:** 2026

**Authors:** Hanieh Taheri, Fatemeh Seydi, Farideh Jalali-Mashayekhi, Azam Moslemi, Heidar Farahani, Ali Khosrowbeygi

**Affiliations:** 1 Student Research Committee, Arak University of Medical Sciences, Arak, Iran; 2 Department of Obstetrics and Gynecology, School of Medicine, Arak University of Medical Sciences, Arak, Iran; 3 Department of Biochemistry and Genetics, School of Medicine, Arak University of Medical Sciences, Arak, Iran; 4 Department of Biostatistics, School of Medicine, Arak University of Medical Sciences, Arak, Iran; 5 Molecular and Medicine Research Center, Arak University of Medical Sciences, Arak, Iran

**Keywords:** Arctium, oxidative stress, inflammation, polycystic ovary syndrome, complementary therapies, randomized controlled trial

## Abstract

**Objective:**

Polycystic ovary syndrome (PCOS) is one of the most prevalent disorders of the endocrine system, with significant implications for female fertility. Antioxidant supplementation may contribute to its better management. This study aimed to investigate the effects of Arctium lappa L. root powder as an antioxidant on some markers of oxidative stress and inflammation, ovary volume, hirsutism score, and menstrual frequency in women with polycystic ovary syndrome.

**Methods:**

This randomized, double-blind, controlled clinical trial was conducted in 2023-2024 at Arak University of Medical Sciences. Sixty subjects with polycystic ovary syndrome were selected by convenience sampling method and allocated to Arctium lappa L. (n=30) and placebo (n=30) by permuted block randomization method and then treated with Arctium lappa L. root powder (460 mg/day) or placebo (460 mg/day) for 12 weeks. Before and after the intervention, some markers of oxidative stress and inflammation, ovary volume, hirsutism score, and menstrual frequency were measured and compared between the two groups.

**Results:**

The values of antioxidative markers, such as superoxide dismutase and catalase, increased significantly (p<0.001). Furthermore, values of oxidative and inflammation markers such as malondialdehyde and C-reactive protein decreased significantly (p<0.001) in the Arctium lappa L. compared to the placebo group. Moreover, the volume of the right and left ovaries was also reduced significantly (p=0.02, p=0.01).

**Conclusions:**

Consuming 460 mg of Arctium lappa L. root powder daily for 12 weeks can reduce ovarian volume and lower oxidative stress and inflammation in women with polycystic ovary syndrome.

**Figure:**
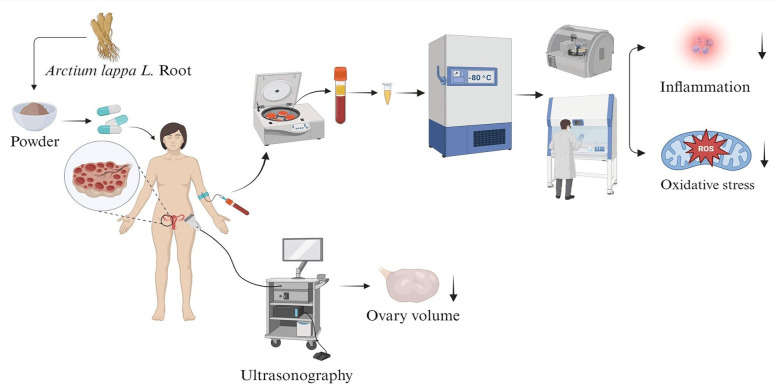

## INTRODUCTION

Polycystic ovary syndrome (PCOS), known as Stein-Leventhal syndrome, is one of the most common diseases related to the endocrine system, characterized by hyperandrogenism, oligo/or anovulation, and polycystic ovaries on ultrasound (Aggarwal & Chakole, 2023). It causes ovarian dysfunction, infertility, endometrial cancer, metabolic syndrome, non-alcoholic fatty liver disease, hypertension, pre-eclampsia, insulin resistance, type 2 diabetes mellitus, gestational diabetes, and cardiovascular diseases in women (Hopkins *et al.*, 2024), and its complications impose a large economic burden on patients, families, and society. Lifestyle modifications, together with medication, are used to treat PCOS (Ma *et al.*, 2022).

Conventional medications such as metformin and clomiphene citrate are associated with certain side effects (Zhou & Hultgren, 2020; Barcroft *et al.*, 2021). Metformin can cause gastrointestinal issues like nausea and diarrhea (Zhou & Hultgren, 2020), and clomiphene citrate use may be associated with an increased risk of ovarian cancer (Barcroft *et al.*, 2021).

Oxidative stress (OS) is the term used to describe the imbalance that exists between oxidants and antioxidants in the body. Reactive oxygen species negatively impact the body due to an imbalance brought on by an overabundance of oxidants. Since OS and inflammation play an essential role in developing PCOS (Sen *et al.*, 2024), antioxidant supplements may be effective in improving the treatment of this disease (Pingarrón Santofímia *et al.*, 2023).

Recently, there has been a growing interest in exploring alternative therapeutic options derived from natural products. *Arctium Lappa L.* (*AL*), known as Burdock, is characterized by purple, spherical flowers, oval leaves, and a non-woody stem. For centuries, plant parts such as roots, leaves, and seeds have been used as home remedies for a wide range of conditions, including infections, sore throats, skin diseases, rashes, boils, tumors, and diabetes. Biochemically, it serves multiple roles, including antimicrobial, antitumor, antioxidant, and anti-inflammatory properties, as well as acting as a modulator of complement system activation. *AL* root has significant antioxidant activity due to the presence of antioxidant compounds such as chlorogenic acid, caffeoylquinic acid, and polysaccharides. The general use of *AL* root is for treating allergies, constipation, and skin diseases (de Souza *et al.*, 2022). Also, it has gastroprotective, hepatoprotective, neuroprotective, and anti-sterility effects (Wang *et al.*, 2016; de Souza *et al.*, 2022). Nevertheless, there has been no investigation of its effects on PCOS in human subjects. Therefore, the current study aimed to evaluate the effects of *AL* root powder on some OS and inflammation biomarkers, ovary volume (OV), hirsutism score, and menstrual frequency in women with PCOS.

## MATERIALS AND METHODS

### Study Design

This randomized, double-blind controlled clinical trial with an allocation ratio of 1:1, was conducted in accordance with CONSORT reporting guidelines. Women with PCOS who were referred to the university’s gynecology clinic from April 2023 to January 2024 and whose disease had been confirmed according to the Rotterdam criteria (Aggarwal & Chakole, 2023) by a specialist were included in the study.

### Inclusion and Exclusion Criteria

The inclusion criteria were women aged 18 to 45 years with a confirmed diagnosis of PCOS according to the Rotterdam criteria by a specialist, subject’s satisfaction with entering the study, and subjects who did not intend to become pregnant. The exclusion criteria were diabetes, thyroid disorders, hyperprolactinemia, pregnancy, breastfeeding, smoking, consumption of antioxidant and anti-inflammatory supplements for three months before the study, and anticoagulant medicines.

### Sample Size

Using reduced glutathione (GSH) as a primary outcome variable, utilizing data from a previous study (Shokrpour & Asemi, 2019) and based on a confidence coefficient of 0.95 and a power of 0.80, with an assumed mean difference (µ_1_ = 483.8 *vs*. µ_2_ = 519.4) and a standard deviation (σ) of approximately 47.7, according to the following equation, the sample size was calculated as ≥28 for each group:


$$n\geq 2\frac{{\left(z_{\alpha }+z_{\beta }\right)}^{2}\sigma^{2}}{{\left({µ}_{1}-{µ}_{2}\right)}^{2}}$$

To account for a potential 10% dropout rate, the final sample size was set at 30 participants per group.

### Randomization

#### Sequence Generation

The permuted block randomization method with blocks of four was employed in this study. The *AL* group was labelled as A, and the placebo group as B. Six possible combinations-AABB, BBAA, BABA, ABBA, BAAB, and ABAB-were written on separate sheets of paper and placed into a container. Each time, one sheet was randomly drawn from the container, the combination on it was recorded, and the sheet was returned to the container. Given the sample size of 60, this process was repeated 15 times, with each combination being recorded sequentially. Subsequently, each individual assignment (letter A or B) in the recorded sequence was assigned a unique number from one to 60.

#### Allocation Concealment Mechanism

Each of these 60 individual assignments (A or B) was placed into a sequentially numbered opaque envelope, and the corresponding number was written on the exterior of the envelope. Whenever a participant was recruited, one envelope was opened in sequence, and based on the letter inside, the subject was assigned to either the *AL* or placebo group.

#### Implementation

A.M. generated the random allocation sequence, F.S. was responsible for enrolling participants, and H.T. performed the assignment of participants to either the *AL* or placebo groups by opening the envelopes.

### Blinding

The study was double-blinded, ensuring that both participants (subjects) and healthcare providers were unaware of the group assignments. To accomplish this, the *AL* and placebo capsules were prepared to be identical in shape, color, and smell, as well as packaged in identical opaque containers. Additionally, the allocation sequence was concealed by placing individual assignments into sequentially numbered, opaque envelopes to ensure that participants were unsure of their treatment assignment until the moment they were assigned and the envelope was opened.

### Intervention Procedures

#### Intervention Dose Selection

As recommended in the “PDR for Herbal Medicines” (Gruenwald *et al.*, 2008) on page 129, 460 mg of the whole dried root powder of *AL* was selected. This book is not merely a traditional text but a systematic documentation of years of empirical observation and clinical experience within phytotherapy. In this publication, dosages that have long been practiced and demonstrated to be effective and safe are codified.

### Intervention Administration

After providing informed consent, participants received either the *AL* or placebo capsules. During the 12-weeks of the study, the *AL* group consumed one capsule (containing 460 mg of *AL* root powder, approved by the Medicinal Plants Department of Arak University), and the placebo group consumed one capsule (containing 460 mg of starch) with lunch daily. To promote adherence and monitor compliance, participants were contacted weekly and reminded to maintain their usual diet and daily activity, to take the assigned capsules, and to report any potential adverse effects or discomfort. At the end of the trial, participants were asked to return the empty containers for pill count assessment. During the study, all participants continued their standard concomitant medications, which included metformin (500 mg) and an oral contraceptive pill (3 mg drospirenone + 0.03 mg ethinyl estradiol).

### Demographic and Anthropometric Data Collection

At the beginning of the study, several demographic and anthropometric factors, such as age, height, and weight, were evaluated by a specialist physician. Body mass index (BMI) was subsequently calculated from these measurements. After the intervention, weight and BMI were re-measured.

### Clinical Assessments

Before and after the intervention, the Modified Ferriman-Gallwey (MFG) score was employed by a specialist physician to quantify the extent of hirsutism. This score ranks the nine body regions, which include the upper lip, chin, chest, upper and lower abdomen, buttocks, upper back, arms, and thighs, from zero (no coarse hairs) to four (frankly virile). The scores are subsequently aggregated to get a “total MFG” score. The total MFG score is the most commonly utilized tool for the clinical evaluation of hirsutism. A total MFG score of ≥8 indicates clinical hirsutism. Mild hirsutism is characterized by scores ranging from 8 to 15, moderate hirsutism from 16 to 25, and severe hirsutism by scores beyond 25 (Willis *et al.*, 2020).

OV was measured using ultrasonography by a single sonologist, who was blinded to the group assignments to ensure consistency. The frequency of menstruation was calculated by dividing the total number of menstruations that occurred in three months by the expected number of menstruations (three times in three months), as assessed by the specialist physician.

### Laboratory Methods

#### Sample Collection and Preparation

Before and after the intervention, following a 12-hour fast, venous blood samples were drawn from the subjects. The blood samples were centrifuged for 10 minutes at 3000 rpm, and the serum samples were stored at -80°C until measurement.

### Biochemical Assays

All biochemical assays were performed by outcome assessors using commercially available kits from Novin Navand Salamat Pishtaz Co. (Urmia, Iran) to evaluate activities of superoxide dismutase (SOD) and glutathione peroxidase (GPx) and levels of GSH and oxidized glutathione (GSSG). Malondialdehyde (MDA) levels were also determined using a commercially available kit from Kushan Zist Azma Parseh Co. (Tehran, Iran). Furthermore, a colorimetric method was used to measure serum levels of fasting blood sugar (FBS), triglyceride (TG), albumin (Alb), uric acid (UA), blood urea nitrogen (BUN), and total bilirubin (Bili), and a turbidimetric method was used for C-reactive protein (CRP) (Delta Darman Part Co., Tehran, Iran) using a chemistry autoanalyzer (Hitachi 717, Japan). Activity of catalase (CAT) and levels of total thiol (TT) were measured using Rezaei et al.’s method (Rezaei Vandchali *et al.*, 2020) and Ellman’s reagent (Wanes *et al.*, 2020), respectively. Total antioxidant capacity (TAC) and total oxidant status (TOS) were evaluated by the ferric reducing ability of plasma (FRAP) assay and the ferric-xylenol orange method (Rezaei Vandchali *et al.*, 2020), respectively. The OS index (OSI = TOS/TAC), CRP/Alb ratio (CAR = CRP/Alb), BUN/Alb ratio (BAR = BUN/Alb), MDA/TAC ratio, and GSH/GSSG ratio were calculated. Total antioxidant gap (TAG), triglyceride glucose index (TyG), and albumin-bilirubin score (ALBI) were calculated by the following formulas:

TAG (µM) = TAC (µM) - [(Albumin (µM) × 0.69) + Uric acid (µM)] (Miller *et al.*, 1997)

TyG Index = ln [Fasting triacylglycerols (mg/dl) × Fasting glucose (mg/dl)/2] (Simental-Mendía *et al.*, 2008)

ALBI = (Albumin (g/l) × -0.085) + (log10 Bilirubin (µM) × 0.66) (Johnson *et al.*, 2015)

### Outcome Measures

#### Primary Outcomes

Oxidative stress markers, including SOD, GPx, GSH, GSSG, MDA, CAT, TT, TAC, and TOS were evaluated, and the OSI, MDA/TAC ratio, GSH/GSSG ratio, and TAG were calculated as primary outcomes.

### Secondary Outcomes

Secondary outcomes included CRP levels, MFG score, OV, and frequency of menstruation. The CAR, BAR, ALBI, and TyG were also calculated and considered secondary outcomes.

### Ethical Consideration

The Arak University of Medical Sciences Ethics Committee approved this randomized double-blind controlled clinical trial (IR.ARAKMU.REC.1401.334) according to the Helsinki ethical statement, and the study was registered with the Iranian Registry of Clinical Trials (IRCT20230222057492N1) in March 2023, and relevant information was updated in May, June, October 2024, and February 2025. All subjects signed a consent form for participation.

### Statistical Analysis

Statistical analysis was conducted using SPSS software (version 23, Chicago, IL, USA). Demographic and anthropometric factors were reported as Mean ± SD, and the results of other variables were presented as Mean ± SEM. A *p*-value of less than 0.05 was considered statistically significant. The Shapiro-Wilk test was utilized to assess the normality of quantitative variables. For comparison of quantitative variables, Paired t-tests were used for within-group changes, while Independent t-tests were employed for between-group comparisons. Additionally, an Analysis of Covariance (ANCOVA) was performed to adjust the baseline differences between the two groups. Furthermore, partial eta square (ηp^2^) was reported as an indicator of effect size.

## RESULTS

**Figure 1:**
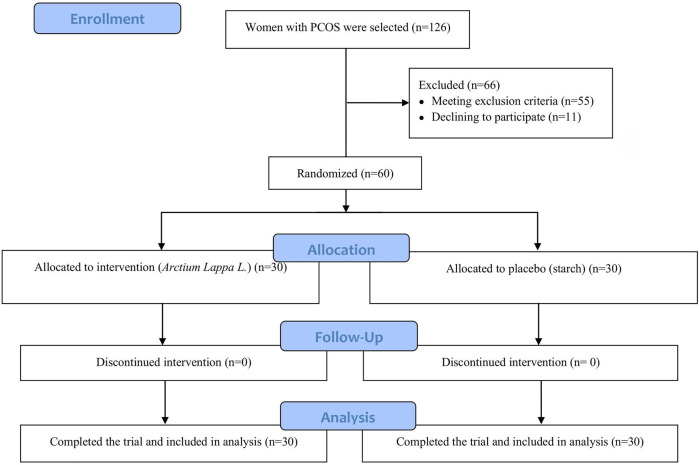
CONSORT flow diagram of the study population .

One hundred twenty-six women with PCOS were selected using convenience sampling. Fifty-five subjects were excluded based on exclusion criteria, and 11 subjects declined to participate, resulting in a final sample of 60 participants. These participants were divided into two groups, *AL* and placebo, using the permuted block randomization method with blocks of four. All 60 participants completed the study without any dropouts, and importantly, no side effects were reported throughout the trial (Fig. 1).

The subjects demographic and anthropometric details are shown in Table 1. No significant differences were observed in the values of age, height, weight, and BMI between the *AL* and the placebo groups before and after the intervention (*p*>0.05).

**Table 1:** Comparison of demographic and anthropometric characteristics of women with PCOS.

Variables	Arctium lappa L. (n=30)	Placebo (n=30)	*p*-value^*^
Age (years)	27.23±5.85	27.77±5.45	0.72
Height (m)	1.64±0.04	1.61±0.05	0.11
Weight before (Kg)	70.13±15.46	68.60±14.14	0.69
Weight after (Kg)	68.73±14.63	67.47±12.40	0.72
BMI before (Kg/m^2^)	26.23±5.93	26.41±5.68	0.91
BMI after (Kg/m^2^)	25.70±5.52	25.98±5.04	0.84

Data presented as Mean ± SD. A significance level of 0.05 was considered (*p*-value < 0.05).

* independent t-test.

Polycystic ovary syndrome (PCOS), Body mass index (BMI).

As shown in Table 2 and Table 3, the study results were compared at baseline and at the end of the trial between and within groups. At the beginning of the study, no significant differences were observed between the two groups in any variable.

**Table 2:** Comparison of the effects of AL and placebo on biochemical characteristics of women with PCOS.

Variables	Arctium lappa L. (n=30)	Placebo (n=30)	*p*-value^*^	*p*-value ^§^	Partial eta square^†^ (ηp^2^)
**SOD (U/ml)** Before After *p*-value^^**^^	339.17±6.34 400.37±15.83 <0.001	324.46±11.04 336.05±6.56 0.39	0.25 <0.001	<0.001	0.20
**CAT (KU)** Before After *p*-value^^**^^	6.15±0.64 8.74±0.47 <0.001	6.40±0.56 6.68±0.39 0.69	0.76 <0.001	<0.001	0.16
**GPx (mU/ml)** Before After *p*-value^^**^^	245.04±2.22 252.57±0.46 <0.001	244.43±2.41 245.33±1.59 0.76	0.85 <0.001	<0.001	0.25
**GSH (µM)** Before After *p*-value^^**^^	6.91±0.51 22.89±1.75 <0.001	6.20±0.96 15.85±2.15 <0.001	0.52 0.01	0.02	0.10
**GSSG (µM)** Before After *p*-value^^**^^	0.447±0.040 0.355±0.030 0.01	0.445±0.037 0.443±0.029 0.96	0.98 0.04	0.02	0.09
GSH/GSSG Before After *p*-value^^**^^	17.56±1.72 76.20±7.51 <0.001	15.09±2.35 43.56±9.52 0.01	0.40 0.01	0.01	0.11
**TAC (mM)** Before After *p*-value^^**^^	0.71±0.03 1.05±0.04 <0.001	0.70±0.03 0.66±0.02 0.23	0.77 <0.001	<0.001	0.56
**TOS (µM)** Before After *p*-value^^**^^	12.19±1.17 8.05±0.62 <0.001	13.23±1 14.21±1.01 0.40	0.50 <0.001	<0.001	0.32
**OSI %** Before After *p*-value^^**^^	1.83±0.13 0.78±0.06 <0.001	2±0.15 2.23±0.17 0.24	0.39 <0.001	<0.001	0.53
MDA (µM) Before After *p*-value^^**^^	4.57±0.47 2.45±0.25 <0.001	4.42±0.58 5.67±0.79 0.26	0.84 <0.001	<0.001	0.21
MDA/TAC Before After *p*-value^**^	0.0066±0.0006 0.0024±0.0002 <0.001	0.0065±0.0009 0.0087±0.0014 0.23	0.95 <0.001	<0.001	0.25
TAG (µM) Before After *p*-value^**^	(-128.06)±29/79 75.67±39/55 <0.001	(-169.53)±29.14 (-242.79)±22.25 0.04	0.32 <0.001	<0.001	0.45
TT (mM) Before After *p*-value^**^	0.157±0.006 0.198±0.009 <0.001	0.169±0.007 0.162±0.004 0.44	0.22 <0.001	<0.001	0.18
**CRP (mg/dl)** Before After *p*-value^^**^^	0.457±0.043 0.310±0.035 <0.001	0.463±0.044 0.447±0.039 0.66	0.91 0.01	<0.001	0.17
**CAR (mg/g)** Before After *p*-value^^**^^	0.099±0.011 0.058±0.007 <0.001	0.097±0.009 0.093±0.008 0.64	0.87 <0.001	<0.001	0.27
**BAR (mg/g)** Before After *p*-value^^**^^	4.82±0.14 2.95±0.12 <0.001	4.87±0.11 4.85±0.10 0.89	0.82 <0.001	<0.001	0.74
**ALBI** Before After *p*-value^^**^^	(-3.46)±0.09 (-4.07)±0.10 <0.001	(-3.41)±0.04 (-3.45)±0.05 0.33	0.63 <0.001	<0.001	0.36
**TyG** Before After *p*-value^^**^^	9.135±0.093 8.768±0.090 <0.001	9.182±0.083 8.999±0.077 <0.001	0.71 0.06	<0.001	0.33

Data presented as Mean ± SEM. A significance level of 0.05 was considered (*p*-value < 0.05).

* independent t-test,

** paired t-test,

§ ANCOVA,

† Partial eta square for ANCOVA. *Arctium lappa L.* (AL), Polycystic ovary syndrome (PCOS), Superoxide dismutase (SOD), Catalase (CAT), Glutathione peroxidase (GPx), Reduced glutathione (GSH), Oxidized glutathione (GSSG), Total antioxidant capacity (TAC), Total oxidant status (TOS), Oxidative stress index (OSI), Malondialdehyde (MDA), Total antioxidant gap (TAG), Total thiol (TT), C-reactive protein (CRP), CRP/albumin ratio (CAR), Bun/albumin ratio (BAR), Albumin-bilirubin score (ALBI), Triglyceride glucose index (TyG).

**Table 3:** Comparison of the effects of *AL* and placebo on clinical characteristics of women with PCOS.

Variables	Arctium lappa L. (n=30)	Placebo (n=30)	p-value^*^	p-value^§^	Partial eta square^†^ (ηp^2^)
**OV. Right (CC)** Before After *p*-value^^**^^	14.65±0.77 11.68±0.53 <0.001	15.02±0.60 13.50±0.68 0.01	0.71 0.04	0.02	0.09
**OV. Left (CC)** Before After *p*-value^^**^^	13.47±0.93 11.55±0.58 <0.001	14.18±0.69 13.38±0.61 0.06	0.54 0.03	0.01	0.12
**MFG Score** Before After *p*-value^^**^^	11.67±0.50 10.30±0.50 0.02	11.07±0.55 11.10±0.62 0.94	0.42 0.32	0.08	0.05
**Menstrual** **frequency** Before After *p*-value^^**^^	0.222±0.068 0.933±0.034 <0.001	0.244±0.084 0.878±0.044 <0.001	0.84 0.32	0.29	0.02

Data presented as Mean ± SEM. A significance level of 0.05 was considered (*p*-value < 0.05).

* independent t-test,

** paired t-test,

§ ANCOVA, †Partial eta square for ANCOVA. *Arctium lappa L.* (AL), Polycystic ovary syndrome (PCOS), Ovary volume right (OV. Right), Ovary volume left (OV. Left), Modified ferriman-gallwey (MFG).

Statistical analysis using independent t-test at the end of the trial revealed that the *AL* group showed significant increases in the following outcomes compared to the placebo group: SOD, CAT, GPx, TAC, TAG, TT, GSH/GSSG, and GSH (all *p*<0.05). Conversely, the *AL* group demonstrated significant decreases in TOS, OSI, MDA, MDA/TAC, BAR, ALBI, CAR, GSSG, CRP, and both left and right ovarian volume (OV) (all *p*<0.05). The full results of the t-test analysis are presented in Tables 2 and 3.

The results of the ANCOVA largely confirmed the findings of the independent t-test. Significant between-group differences favoring the *AL* group were observed for all mentioned biochemical and inflammatory markers, as well as for left and right ovarian volume (OV) (all *p*<0.05). Notably, after adjusting for baseline values, the TyG index showed a significant decrease in the *AL* group compared to the placebo group (*p*<0.05), a difference that was not significant in the independent t-test. However, the ANCOVA for MFG score and menstrual frequency remained non-significant. The effect sizes (ηp^2^ for ANCOVA) for all reported variables are presented in detail in Tables 2 and 3.

## DISCUSSION

The results of current study demonstrated that the daily consumption of *AL* root powder for 12-weeks increased antioxidative markers while decreasing oxidative and inflammatory markers. Additionally, it improved OV in PCOS women.

Various studies have shown that levels of OS are higher in women with PCOS (Sen *et al.*, 2024). Therefore, we evaluated some OS markers to assess the effect of *AL* on OS in affected women. *AL* root has been shown to protect against some conditions such as, atherosclerosis, knee osteoarthritis, acute lung injury, and diabetes by reducing OS. This is achieved by decreasing MDA levels and increasing SOD, CAT, TAC, TT, GPx, and GSH levels. Moreover, it improves diabetes-induced changes in behavioral indicators (Maghsoumi-Norouzabad *et al.*, 2016; Wang *et al.*, 2016; Hosseini Arya *et al.*, 2023; Lu *et al.*, 2023). The antioxidant properties of *AL* root are due to the presence of various groups and chemical compounds, including quercetin (de Souza *et al.*, 2022). For instance, one study demonstrated that using quercetin as a supplement for 30 days reduced oxidative damage caused by iron oxide nanoparticles by enhancing levels of GSH and GSH/GSSG in mouse brain tissue (Dora *et al.*, 2021). Similarly, another study found that a 24-hour treatment of hepatocytes with varying doses of quercetin can mitigate ethanol-induced liver damage by significantly increasing TAC and decreasing TOS and OSI levels (Zhao *et al.*, 2022). Also, another important OS biomarker is TAG (Mandani *et al.*, 2021). Alpha-lipoic acid supplementation, as an antioxidant, for eight weeks resulted in a significant increase in serum TAG level and a decrease in FBS level in women with gestational diabetes (Mandani *et al.*, 2021). Consistent with these findings, the results of the current study suggest that *AL* root can reduce OS and benefit individuals with PCOS. This may be due to the presence of polyphenols, flavonoids, and lignans in the root, which combat free radicals and exhibit potent antioxidant properties (de Souza *et al.*, 2022).

CRP, CAR, and BAR are inflammatory indicators that have a positive correlation with the severity of diseases (Dey *et al.*, 2023; Kaeley *et al.*, 2023; Uzum & Turkkan, 2023). A study showed that *AL* root tea significantly reduced serum CRP levels in patients with knee osteoarthritis due to its antioxidant effects. Similarly, another study claimed that consumption of Spirulina platensis capsules, as an antioxidant, causes a significant decrease in BAR in patients with COVID-19 (Maghsoumi-Norouzabad *et al.*, 2016; Hatami *et al.*, 2023). These results, similar to the present study, show that the consumption of antioxidant supplements such as *AL* root can reduce the mentioned inflammatory indicators and subsequently reduce the severity of the diseases. It was reported that quercetin in *AL* root can inhibit the nuclear factor kappa-light-chain-enhancer of activated B cells pathway, decreasing the expression of inflammatory cytokines and, conversely, increasing the expression of anti-inflammatory cytokines (Ren *et al.*, 2019).

The ALBI score evaluates the level of liver dysfunction and provides valuable information about the disease’s prognosis. Patients with a lower score have a better prognosis (Sungur *et al.*, 2024). A research study demonstrated a negative correlation between plasma omega-3 fatty acids levels and the ALBI score in patients with liver cirrhosis (Sano *et al.*, 2024). The results of this study, along with the current research, showed that increasing the amount of serum antioxidants such as *AL* root causes a decrease in the ALBI score and improves liver function in subjects. On the other hand, the reduction of this score in the *AL* group compared to the placebo group indicates that the daily consumption of 460 mg of *AL* root powder not only didn’t cause liver toxicity in the *AL* group but also improved the liver condition of these subjects compared to the placebo group.

The TyG index is a marker that can be used to predict insulin resistance and is linked to the development of type 2 diabetes and metabolic syndrome (Kwon *et al.*, 2023). Consumption of lyophilized onion powder, rich in quercetin, causes a significant decrease in the TyG index, insulin resistance, and glucose in an animal model of obesity (Balderas *et al.*, 2022). Analogously, results of the current study indicated that consumption of *AL* root powder reduces the TyG index and probably lowers the risk of type 2 diabetes and metabolic syndrome in women with PCOS.

Oligomenorrhea and amenorrhea are clinical symptoms of PCOS (Aggarwal & Chakole, 2023) and an MFG score of more than eight indicates hirsutism in them (Willis *et al.*, 2020). Furthermore, increased OV is one of the clinical indicators of PCOS (Hopkins *et al.*, 2024). A study stated that the daily intake of a combined supplement of alpha-lipoic acid and myoinositol for six months causes a significant increase in the number of menstrual cycles in women with PCOS (De Cicco *et al.*, 2017). Other study found that consuming a combined supplement of alpha-lipoic acid, N-acetyl cysteine, vitamin B6, and S-Adenosyl methionine for six months significantly reduces the MFG score in women with PCOS (Pingarrón Santofímia *et al.*, 2023), and another study showed that resveratrol supplementation for three months causes a significant decrease in OV in them (Hashemi Taheri *et al.*, 2022).

Consistent with Hashemi Taheri et al. findings, our study’s results showed a significant reduction in OV in the intervention group compared to the placebo group. This suggests that *AL* root, likely due to its antioxidant properties, can effectively improve this specific clinical feature of PCOS.

However, despite the significant improvement in OV, our results did not show a significant difference between the intervention and placebo groups in menstrual frequency and MFG score. These outcomes may be attributed to the relatively small sample size or the study’s duration, factors that may have limited our ability to detect a significant between-group effect for menstrual frequency and hirsutism.

In conclusion, our findings suggest that *AL* root, as a potent antioxidant, can alleviate some PCOS symptoms, particularly OV, by mitigating OS, inflammation, and insulin resistance, thereby offering a promising therapeutic approach for managing this syndrome.

### Propose Mechanism

The proposed mechanism for these effects is that quercetin diminishes insulin resistance by activating the AMP-activated protein kinase signaling pathway (Ren *et al.*, 2019) and decreases androgen levels by regulating the activity of the rate-limiting enzyme, CYP17A1, of the hormone synthesis pathway. In addition, quercetin can rectify aberrant levels of LH/FSH by influencing the ovarian-pituitary axis (Su *et al.*, 2024).

### Safety Profile

According to the “PDR for Herbal Medicines” (p. 129), there is a slight possibility of sensitivity reactions following skin contact with *AL*; however, no side effects have been reported when it is consumed at appropriate therapeutic doses (Gruenwald *et al.*, 2008). In our study, none of the participants reported any side effects during the 12-week intervention period. Similarly, a randomized clinical trial published in 2018 explicitly stated that the consumption of an herbal formulation containing *AL* in subjects with Helicobacter pylori infection did not result in any adverse effects (Yen *et al.*, 2018). Furthermore, two additional clinical trials, one in 2018 investigating the effects of *AL* root extract in older Korean women (Ha *et al.*, 2018) and another in 2016 examining *AL* root tea in subjects with knee osteoarthritis (Maghsoumi-Norouzabad *et al.*, 2016), did not specifically mention the absence of adverse events but also did not document any notable side effects. Collectively, these findings suggest that *AL* root is generally safe and well-tolerated when consumed within the recommended therapeutic range.

### Strengths and limitations

The limitations of this study were the short treatment period for the subjects and the impossibility of measuring the expression of effective genes in this syndrome. However, a significant strength was finding substantial effect sizes for several key outcomes. While the exact magnitude of these effects needs further validation, their prominence underscores the considerable practical and clinical importance of *AL* supplementation. We suggest that future clinical trials with a longer duration and different doses of *AL* root powder and long-term follow-up of the subjects should be conducted; if possible, the expression of genes effective in PCOS might be investigated.

## CONCLUSION

According to the findings of the current study, consuming 460 mg of *AL* root powder daily for 12 weeks can reduce OV and lower oxidative stress and inflammation in women with PCOS.

These results suggest that *AL* root may represent a promising complementary therapeutic approach for managing this syndrome, pending further validation.
